# Trophic niche similarities of sympatric *Turdus* thrushes determined by fecal contents, stable isotopes, and bipartite network approaches

**DOI:** 10.1002/ece3.6485

**Published:** 2020-08-17

**Authors:** Camila Bosenbecker, Leandro Bugoni

**Affiliations:** ^1^ Laboratório de Aves Aquáticas e Tartarugas Marinhas (LAATM) Instituto de Ciências Biológicas & Programa de Pós‐Graduação em Biologia de Ambientes Aquáticos Continentais Universidade Federal do Rio Grande – FURG Rio Grande Brazil

**Keywords:** bipartite network analysis, diet, ecological niche, feeding ecology, niche partitioning, stable isotope analysis

## Abstract

An ecological niche has been defined as an *n*‐dimensional hypervolume formed by conditions and resources that species need to survive, grow, and reproduce. In practice, such niche dimensions are measurable and describe how species share resources, which has been thought to be a crucial mechanism for coexistence and a major driver of broad biodiversity patterns. Here, we investigate resource partitioning and trophic interactions of three sympatric, phylogenetically related and morphologically similar species of thrushes (*Turdus* spp.). Based on one year of data collected in southern Brazil, we investigated niche partitioning using three approaches: diet and trophic niche assessed by fecal analysis, diet and niche estimated by stable isotopes in blood and mixing models, and bipartite network analysis derived from direct diet and mixing model outputs. Approaches revealed that the three sympatric thrushes are generalists that feed on similar diets, demonstrating high niche overlap. Fruits from C3 plants were one of the most important food items in their networks, with wide links connecting the three thrush species. *Turdus amaurochalinus* and *T. albicollis* had the greatest trophic and isotopic niche overlap, with 90% and 20% overlap, respectively. There was partitioning of key resources between these two species, with a shared preference for fig tree fruits—*Ficus cestrifolia* (*T. amaurochalinus* PSIRI% = 11.3 and *T. albicollis* = 11.5), which was not present in the diet of *T. rufiventris*. Results added a new approach to the network analysis based on values from the stable isotope mixing models, allowing comparisons between traditional dietary analysis and diet inferred by isotopic mixing models, which reflects food items effectively assimilated in consumer tissues. Both are visualized in bipartite networks and show food‐consumers link strengths. This approach could be useful to other studies using stable isotopes coupled to network analysis, particularly useful in sympatric species with similar niches.

## Graphical abstract

Niche dimensions are measurable and describe how species share resources, which has been thought to be a crucial mechanism for coexistence and a major driver of broad biodiversity patterns. Morphologically, similar species of thrushes (*Turdus* spp.) are generalist species, but distinct, despite overlapping feeding habits allow coexistente.

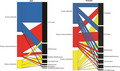

## 1 INTRODUCTION

The ecological niche concept currently used, as developed by Hutchinson (1957), is defined as an *n*‐dimensional hypervolume formed by conditions and resources that species need to survive and breed, addressing their ecological demands. During the development of the concept, the principle of competitive exclusion established that two species with identical niches cannot coexist (Gause, 1934). However, closely related evolutionary lineages tend to be morphologically similar, resulting in species with convergent strategies to cope with their environment, and thus, they usually have analogous ecological requirements. Notwithstanding, there is a maximum limit to the niche overlap among species that allows their coexistence (MacArthur & Levins, 1967). To reduce niche overlap, species usually exploit resources in slightly different ways (Schoener, 1974) through different foraging modes (Mohd‐Azlan, Noske, & Lawes, 2015) or consumption of different resources (Cherel et al., 2008). Where resources are overabundant, competition can be relaxed with species sharing a large proportion of the resource, and thus, overlap can be much larger (Colwell & Futuyma, 1971). Throughout the use of the niche concept in ecology, the trophic niche, the resources that are consumed by a species, has been one of the most extensively studied.

In birds, trophic niches are typically studied using fecal analysis (e.g., Gasperin & Pizo, 2009), pellet analysis (e.g., Silva‐Costa & Bugoni, 2013), gastrointestinal contents of dead birds (Foster, 1987; Mallet‐Rodrigues, 2010) and direct observations or video recordings (Dong, Lu, Zhong, & Yang, 2016). Despite the low cost, rapid data collection, accurate species identification (often to species level), and possibility of quantifying prey size, these methodologies only provide information on recent food items (<60 min in thrushes, Gasperin & Pizo, 2012) and often do not provide information about easily digestible foods, thus limiting or potentially biasing the resulting diet composition (Barrett et al., 2007; Ralph, Nagata, & Ralph, 1985). To overcome such limitations, other techniques to complement dietary analysis have been used, such as molecular‐based methods in feces or stomach contents (e.g., Deagle et al., 2005) or intrinsic markers in consumer tissues, such as stable isotope analysis (Barrett et al., 2007). Through the stable isotope analysis, one may evaluate the composition of the diet of a consumer during the period of tissue synthesis, according to its metabolic activity at different time scales (Dalerum & Angerbjörn, 2005; Kelly, 2000). For whole blood in passerines, the ^13^C half‐life ranges from 5 to 19 days (review in Hahn, Hoye, Korthals, & Klaassen, 2012), which provide a longer time window of food resources used. From different elements, it is possible to calculate an isotope niche space (δ‐space), which dimensions are the elements for which isotope values have been quantified, for example, on the habitat (δ^13^C), trophic level (δ^15^N), and resources consumed (both elements) (Newsome, Martinez, Bearhop, & Phillips, 2007). Through this technique, it is possible to evaluate eventual coexistence between sympatric species (Bodey, Ward, Phillips, McGill, & Bearhop, 2014) and thus analyze the breadth and overlap of isotopic niches (Costa‐Pereira, Araújo, Souza, & Ingram, 2019; Hobson, 2011; Jackson, Inger, Parnell, & Bearhop, 2011), which are proxies for trophic niches (Marshall et al., 2019).

To evaluate the structure of the trophic relationships of coexisting species, different approaches are used based on data from diet analysis, stable isotope analysis, and trophic interaction networks. Trophic interaction network allows evaluating the similarity in links between coexisting species and the relation with their food sources through bipartite matrices, and thus the direct quantification of niche partitioning and complementary specialization (Blüthgen, Menzel, & Blüthgen, 2006). Furthermore, the network structure helps to identify patterns of grouping between species and their resources and to evaluate the role of consumers according to their dietary specialization or generalization in the network (Bascompte, Jordano, Melian, & Olesen, 2003), which sometimes are uncovered by other data analytical tools. The use of isotopes coupled to network analysis is quite recent (Dalerum et al., 2016; Miranda, Dalerum, & Parrini, 2014) and thus is still being fully developed. The approach used here can provide a comparison between diet and isotopes in a way that has not been done yet, that is, using output of isotopic mixing models to depict bipartite networks, instead of previous studies based on raw stable isotope values (Dalerum et al., 2016; Miranda et al., 2014). We predict strong sharing of resources among the three consumers studied, but with marked differences in proportions of food items used (i.e., links strength), as a proxy of niche partitioning.

Thrushes (*Turdus* spp.) are birds that form an abundant and speciose group in the Turdidae family (Collar, 2017). In Brazil, 15 species of this genus have been recorded (Piacentini et al., 2015), while in Rio Grande do Sul state, southern Brazil, there are 6 *Turdus* species (Franz, Agne, Bencke, Bugoni, & Dias, 2018). Thrushes stand out for omnivorous and generalist food habits, often feeding on fruits and seeds, as well as small invertebrates (Manhães, Loures‐Ribeiro, & Dias, 2010; Snow & Snow, 1963). Recent studies have shown that thrush diets are composed of a large variety of resources, with an omnivorous habit but with a predominance of fruits (Durães & Marini, 2005; Gomes, Loiselle, & Alves, 2008; Sabino, Morais, & Duca, 2017; Vogel, Zawadzki, & Metri, 2011). Thrushes are able to occupy different environments, such as fragmented and human‐altered patches (D'Avila, Gomes, Canary, & Bugoni, 2010; da Silveira, Niebuhr, Muylaert, Ribeiro, & Pizo, 2016), and are highly vagile, with some migratory species (Nagy, Végvári, & Varga, 2019). Due to these morphological similarities, food habits, and the frequent coexistence of multiple thrush species in most communities throughout the world, this group makes up a promising study system to advance the understanding of isotopic trophic niche differentiation and coexistence between morphologically similar species.

In this context, we investigated three coexisting thrush species *Turdus amaurochalinus* (Creamy‐bellied Thrush), *Turdus albicollis* (White‐necked Thrush), and *Turdus rufiventris* (Rufous‐bellied Thrush), mean body mass in the area from 64 to 69 g (Rodrigues et al., 2019) to understand patterns of resource partitioning and trophic interactions of sympatric, phylogenetically related and morphologically similar species. Specifically, this study aims to evaluate isotopic trophic niche differentiation and food resource partitioning by thrushes in southern Brazil. We hypothesize the existence of high trophic and isotopic niche overlap among species, and a nested pattern for the two consumer‐resource networks based on traditional (feces) and assimilated (stable isotope analysis) diets, as they rely on similar resources.

## 2 MATERIALS AND METHODS

### 2.1 Study area

**FIGURE 1:**
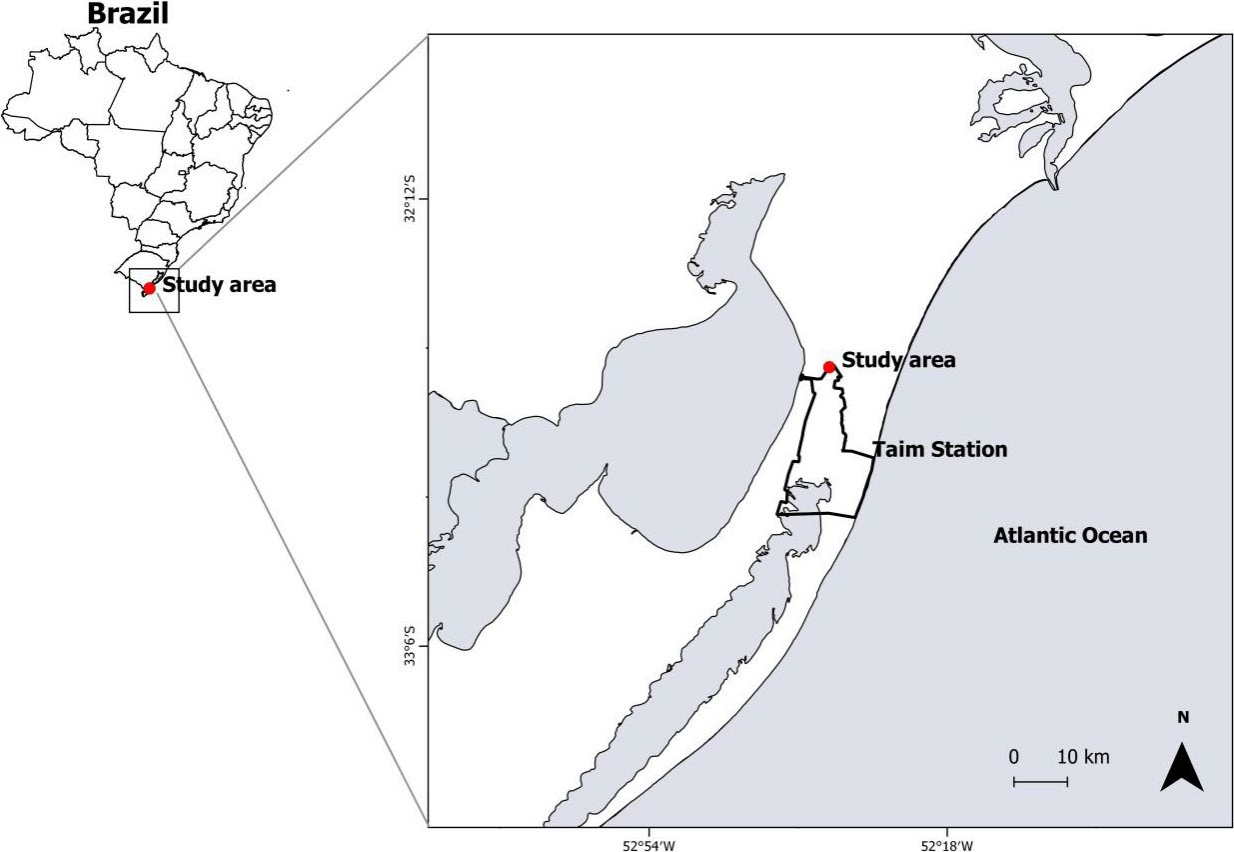
Study area (red dot) at the Taim Ecological Reserve (delimitation ESEC), located in the coastal plain of Rio Grande do Sul, southern Brazil where thrushes were captured

This study was conducted at the Taim Ecological Reserve, a 32,806 ha area, at 32.5°S; 52°W in the southern Brazilian coastal plains. This area is part of the Atlantic Forest Biosphere Reserve (UNESCO, 1998) and a “Ramsar site” by the Convention on Wetlands of International Importance, which recognized it as a key place of biodiversity (Ramsar, 2013). It is located between Mirim and Mangueira Lagoons and the Atlantic Ocean (Waechter, 1985) (Figure 1). The area includes a system of lagoons interconnected by large wetlands and extensive dune regions, *restinga* forests, grasslands, and swamp forests (Waechter, 1985). *Restinga* forests are over sandy soils with the presence of cactuses, while swamp forests have mainly fig trees (*Ficus* spp.) and cork trees (*Erythrina crista‐galli*) (Waechter, 1985). The soil is geologically recent, resulting from a marine deposition process, with accumulation of organic matter in depressions carried by lacustrine waters and wind (Ferreira, Castilho, Hasenack, & Ferraro, 1995). Climatic characteristics with the accumulation of organic matter over the soil and flat terrain result in water clogging in swamp forests (Marques et al., 2013). The climate in the region is subtropical‐humid with an annual average temperature between 13 and 24°C and annual rainfall of 1,200–1,500 mm (Klein, 1998) with the absence of a dry season.

Data collection was restricted to in or around naturally fragmented forest patches, including *restinga* forests and swamp forests (Morellato & Haddad, 2000; Ribeiro, Metzger, Martensen, Ponzoni, & Hirota, 2009), in lowland areas near the coast.

### 2.2 Diet and blood sampling and analysis

Samples of the three species of interest, *T. rufiventris*, *T. albicollis*, and *T. amaurochalinus*, were collected during monthly expeditions from September 2016 to September 2017, lasting 2 days and with a total of 288 h of netting. Birds were captured with 6 mist nets, 36 mm mesh size (12 m × 2.6 m), settled in locations adjacent or inside forest patches, in shadow areas, where intense transit of birds of interest had been observed. Nets were open 30 min before sunrise and closed 30 min after sunset. Captured birds were identified based on Narosky and Yzurieta (2012) and placed in cotton bags for 15 min for the collection of feces and regurgitated material (Sarmento, Alves‐Costa, Ayub, & Mello, 2014). To avoid contamination of samples, bags were replaced for each bird sampled. The feeding ecology of the birds was assessed by two methodologies: one based on fresh feces of the mist netted birds for the analysis of diet composition, which reflects recent (<1 h) feeding by birds, and the other was a stable isotope analysis of the whole blood, which allows the detection of food sources ingested and assimilated (Peterson & Fry, 1987) days or weeks before (Hahn et al., 2012).

Fecal samples were individually stored in vials with 70% ethanol. A total of 100–200 µl of blood from the brachial or tarsal vein of birds were collected with a syringe and needle (4 mm), stored in plastic vials, and frozen. To avoid resampling, individuals were marked with individually coded aluminum bands provided by CEMAVE/ICMBio, the Brazilian Banding Agency.

Potential food sources (fruits and invertebrates) were manually collected based on observations of birds foraging in the area or food items present in the feces. These items were frozen for later stable isotope analysis and were also used as a reference for the identification of the fragments in the fecal samples.

In the laboratory, feces and potential food items were screened in a stereomicroscope, identified at the lowest taxonomic level possible using identification guides (e.g., Carrano‐Moreira, 2015) and consultations with experts. Food items were first counted individually, and then, their reconstituted volume was estimated based on the average volume of items of similar size in a reference collection. To quantify the items present in the diet, the volumetric method was used following Hellawell and Abel (1971) that was modified by Bastos, Miranda, and Garcia (2013). With a millimetric paper under a petri dish, food items were compacted with glass slides, forming blocks of 1 mm^3^.

For the stable isotope analysis, sugar from the potential fruits was extracted using acetone for the elimination of sugars and carotenoids that affect isotopic values (Rossmann, Koziet, Martin, & Dennis, 1997). Blood samples and potential sources were lyophilized for 8 h, homogenized, weighed, and placed in sterilized tin capsules (5 × 9 mm). Samples were analyzed in isotope ratio mass spectrometer at the Stable Isotope Center at the University of New Mexico (UNM) in the USA.

To determine the isotopic values of consumers and their foods, the isotopic ratio (R) of each element in the samples (^13^C/^12^C and ^15^N/^14^N), represented in delta notation (δ) and expressed in parts per thousand (‰), was obtained. Atmospheric air and Vienna Pee Dee Belemnite (VPDB) were the international standards for N and C, respectively, using the equation in Bond and Hobson (2012):

(1) $$\delta^{13}C\;\text{or}\;\delta^{15}N\left(‰\right)=\left(\frac{R_{\text{sample}}}{R_{\text{standard}}}\right)-1$$

Isotopic reference materials were interspersed with samples for drift calibration, and the precision of the measurements was 0.08‰ and 0.03‰ for δ^15^N and δ^13^C, respectively.

### 2.3 Data analysis

From the quantification of the food items present for each thrush species, the following was determined: (a) the frequency of occurrence (FO), as the proportion of samples in which the food item was present; (b) the relative frequency of occurrence (FO%), that is, FO as the percentage of the total number of samples examined; (c) the numerical contribution of each item recorded in the feces (*N*); (d) the numerical proportion of food items in the diet (*N*%); and (e) the prey‐specific numeric contribution (PN%), when only samples in which a given food item occurred were used in calculations. The total volume of each prey or fruit in the food item category (*V*), as well as the relative proportion by volume contribution of the food item, in relation to the total volume of the sample (*V*%), was determined; relative prey‐specific volume contribution (PV%) was determined using only samples in which a given food item occurred. The prey‐specific relative importance index (PSIRI%) using these parameters was calculated according to Brown, Bizzarro, and Ebert (2012), but PM% was changed to PV%.

(2) $$\text{PSIRI}\%=\left[\frac{(\text{PN}\%+\text{PV}\%)\times \text{FO}\%}{2}\right]/100$$

The food niche breadth of each species was calculated using Levins' (1968) index:

(3) $$B=\frac{1}{\Sigma pi^{2}}$$

where *B* is the Levins' measure of the niche breadth; *pi* is the proportion of food items belonging to food category *i*, standardized as.

(4) $${\hat{B}}_{A}=\frac{\hat{B}-1}{n-1}$$

where ${\hat{B}}_{A}$ is the Levins' standardized niche breadth index; $\hat{B}$ is Levins' measure of niche breadth; and *n* is the number of food items (Hurlbert, 1978). Standardized niche breadth is thus expressed on a scale from 0 to 1.

The trophic niche overlap between the species was calculated using Pianka's (1973) index.

(5) $$O_{\mathrm{kj}}=\frac{\sum_{i}^{n}p_{\mathrm{ij}}p_{\mathrm{ik}}}{\sqrt{\sum_{i}^{n}p_{\mathrm{ij}}^{2}\sum_{i}^{n}p_{\mathrm{ik}}^{2}}}$$

where *O_kj_* is Pianka's overlap between species *k* and *j*; *p_ij_* represents the proportion of resource *i* that is used by species *j*; *p_ik_* = the proportion of resource *i* that is used by species *k*; and *n* represents the total number of food items.

In addition, the dietary data for each species were used to classify the predominant and secondary diet types (or trophic guild) of each thrush species, as proposed by Lopes, Fernandes, Medeiros, and Marini (2016).

Stable isotope Bayesian ellipses in R (SIBER package) were used to calculate the isotopic niche of the three thrush species through the area of the standard ellipse adjusted for small sample sizes (SEAc). This output is the niche size of every species and the percentage of overlap between species (Jackson et al., 2011).

To determine the contribution of the different dietary sources to the diet of each species, we used Bayesian stable isotope mixing models in the R package SIAR (Parnell, Inger, Bearhop, & Jackson, 2010). The selection of the sources used in the model was based on the food items found in the feces and information on foraging and diet available from the literature. Sources were grouped by similarity according to their ecological characteristics and isotopic values and after a visual graphical evaluation, as suggested by Post (2002). Samples of potential food items were classified into five food groups: arthropods that were grouped into four different guilds (omnivorous, predatory, herbivorous, terrestrial‐detritivorous, and surface‐detritivorous) and fruits (C3 and C4/CAM photosynthetic pathways) (Table S2). The trophic discrimination factors (TDFs) used in the models were based on a controlled experiment with the omnivorous (insectivorous‐frugivorous) passerine with body mass 35–50 g, the Red‐Throated Ant Tanager *Habia fuscicauda* (2.6 ± 0.2‰ for δ^15^N and 2.2 ± 0.1‰ for δ^13^C) (Herrera & Reyna, 2007). Ideally, the trophic discrimination factors should be from the same species or close taxon. Due to the lack of TDF values for thrushes, we also run analyses with larger *SD* values (2.6 ± 1.0‰ for δ^15^N and 2.2 ± 0.9‰ for δ^13^C), with the aim of encompassing potential sources and thus capture real TDF values within the range.

Due to the low number of samples collected for *T. rufiventris*, a bootstrap resampling method was used to standardize *n* = 50 for each species. Then, bootstrapped stable isotope values were used for calculation of isotopic niche, mixing models, and the isotopic network analysis. To analyze the structural patterns of the trophic interactions of each thrush species with their food sources, we built bipartite networks, considering each thrush species and their food items, in groups as mentioned above. Two weighted matrices were constructed, the first using PSIRI% values of food items in the feces and the second using the mean of contribution the data generated by the output for each isotopic source used in the isotopic mixing models (mean of the 95% credible intervals). This created two consumer‐resource networks, allowing a comparison between species and approaches, that is, isotopic versus dietary. In the networks, the nodes represent the species, the links represent the consumption of a given resource by a species, and the link thickness represents the intensity of consumption. To test the degree of interaction of *Turdus* species specialization, the standardized Kullback–Leibler distance (*d′*) was used (Blüthgen et al., 2006). This index quantifies the difference between two probability distributions and can be used to analyze species‐level interaction specialization variations in networks, ranging from 0 (most generalized) to 1 (most specialized) (Blüthgen et al., 2006).

In order to test whether the diet of certain species is subsets of the diet of other species, we tested the matrices for nestedness, using the WNODF metric, which calculates the nonoverlap and decreasing fill of the matrix for weighted data (Almeida‐Neto & Ulrich, 2011). The observed values of WNODF were compared with those obtained from the null model with 1,000 randomizations, to access metric significances. The null model used was “vaznull,” which converts the original data to a binary matrix and randomizes interactions, maintaining the marginal totals and the connectivity of the original matrix (Vázquez et al., 2007). Nestedness was regarded as significant when the observed value was higher than the 95% confidence interval expected by the null models. All analyses were run in R package “bipartite.”

## 3 RESULTS

A total of 217 food items were identified in the 50 fecal samples of the three thrush species belonging to 18 taxa identified at different levels, including diverse arthropods, C3 and C4 plants (Table S1). There was a wide variety of food resources in the diets, such as arachnids (scorpions and spiders), terrestrial crustaceans, different orders of insects (ants, beetles, and termites), and fruits (*Ficus cestrifolia*, *Opuntia vulgaris*, and *Myrsine* sp.). In comparison with the lower occurrence of arthropods (FO = 69.2%, 45.0%, and 50.0% for *T. amaurochalinus*, *T. albicollis,* and *T. rufiventris*, respectively), plants were present in over 85% of the samples of the three species. However, the largest contribution in terms of number varied according to thrush species, with *Myrsine* sp. (*N*% = 20.0) for *T. amaurochalinus*, Hymenoptera (*N*% = 29.1) for *T. albicollis* and unidentified seeds (*N*% = 42.1) for *T. rufiventris* (Table S1). The prey‐specific index of relative importance (PSIRI%) demonstrated unidentified plant fragments as the most important items for *T. amaurochalinus* and *T. rufiventris* (PSIRI% = 13.4 and 28.1, respectively), while the fruit *F. cestrifolia* was the most important item for *T. albicollis* (PSIRI% = 13.5).

Based on the proportions of food items in the diet, *T. amaurochalinus* and *T. rufiventris* were classified as frugivorous secondarily insectivorous (FR_IN_), as both species had diets containing a >35% fruit volume in relation to the total, and secondarily insectivorous, as they consumed insects between 10% and 35% of their volume. *Turdus albicollis* was classified as frugivorous only (FR) because insects were present in its diet in a much lower proportion, that is, only 6.1% of the volume.

The trophic niche width, measured by standardized Levins' index, was ${\hat{B}}_{A}$ = 0.38 for *T. amaurochalinus*, ${\hat{B}}_{A}$ = 0.35 for *T. albicollis,* and ${\hat{B}}_{A}$ = 0.34 for *T. rufiventris*. The Pianka index indicated a high diet overlap (>0.6) among the species. The largest overlap was found between *T. amaurochalinus* × *T. albicollis* (*O* = 0.9), followed by *T. amaurochalinus* × *T. rufiventris* (*O* = 0.7) and *T. albicollis* × *T. rufiventris*, with *O* = 0.6.

**FIGURE 2:**
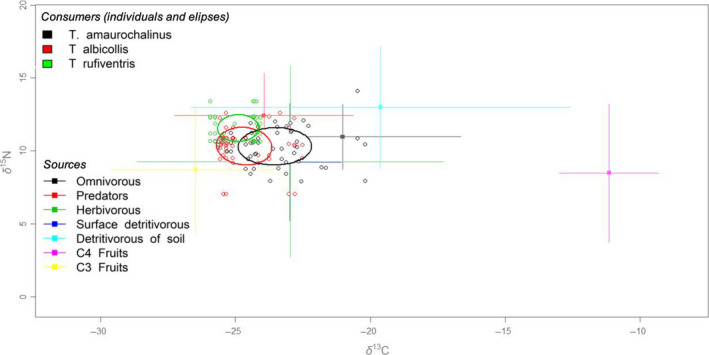
Isotopic niche of the three *Turdus* species in the delta space (δ), based on areas of standard corrected ellipses (SEAc). Values of δ^15^N and δ^13^C (‰) of the potential food items (solid symbols) and isotopic values in the blood of the *Turdus* spp. sampled at the Taim Ecological Reserve (empty symbols). Source values were corrected with a source‐consumer discrimination factor of 2.6‰ for δ^15^N and 2.2‰ for δ^13^C

The nitrogen isotopic values (δ^15^N) in the blood of the birds had a mean ± *SD* from 10.0 ± 1.5‰ for *T. amaurochalinus* to 11.6 ± 1.0 for *T. rufiventris*, with the value for *T. albicollis* in between these values (10.4 ± 1.3‰). For carbon (δ^13^C), the values varied from −25.0 ± 0.7‰ for *T. rufiventris* to −23.4 ± 1.5‰ for *T. amaurochalinus* (Figure 2, Table S2). Thus, the δ^13^C and δ^15^N values, from highest to lowest, followed opposite patterns among the three species. The species with the highest amplitude of its isotopic niche, based on bootstrapped values, was *T. amaurochalinus* (SEAc = 5.5), followed by that of *T. albicollis* (SEAc = 4.2) and *T. rufiventris* (SEAc = 2.2, Figure 2). The highest isotopic niche overlap was between *T. amaurochalinus* × *T. albicollis* (20%), followed by that of *T. albicollis* × *T. rufiventris* (6%). The lowest overlap was <1% among *T. amaurochalinus* × *T. rufiventris*, indicating minimal overlap in the isotopic niche (Figure 2).

**FIGURE 3:**
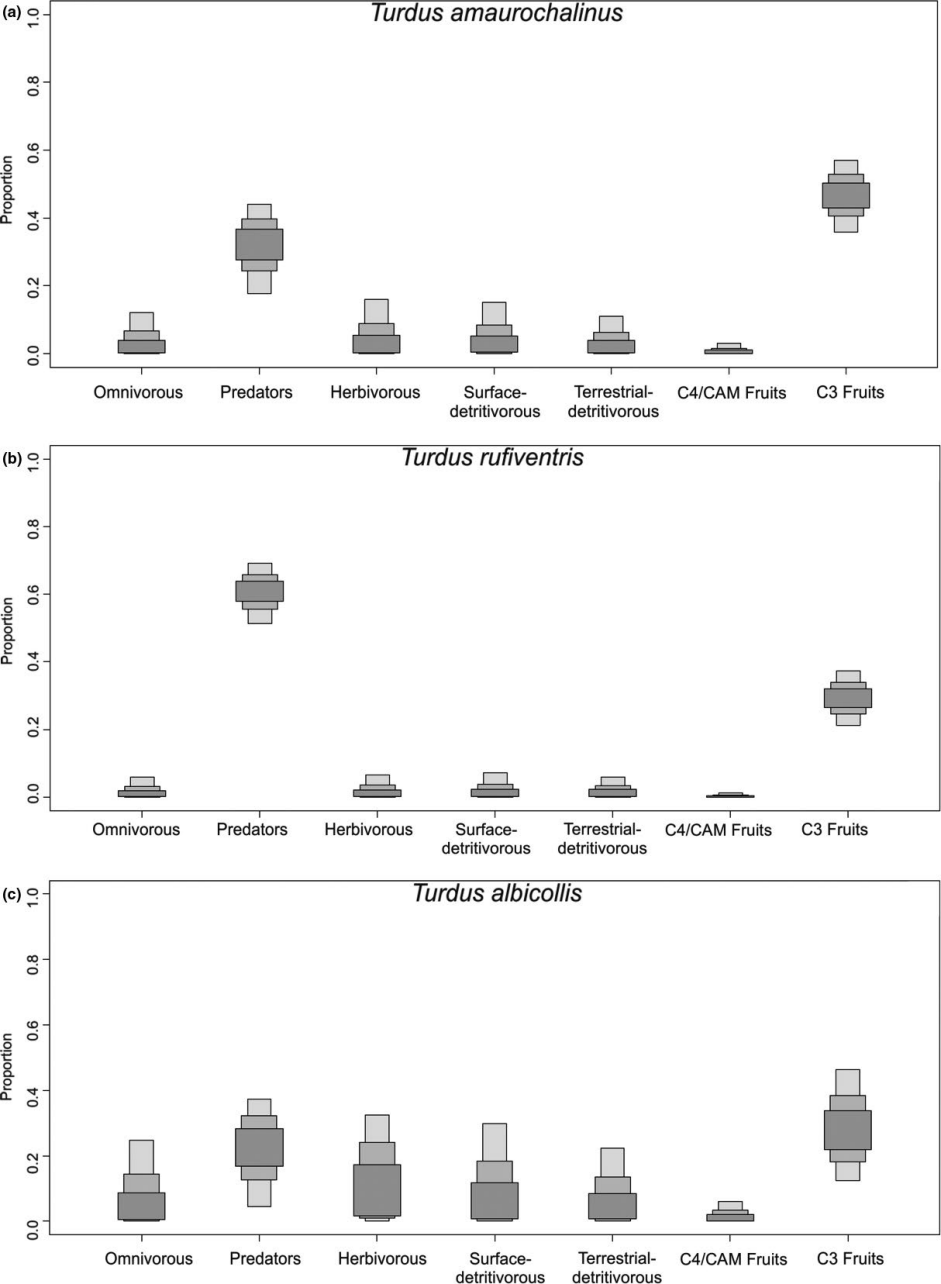
Contribution of different food sources (arthropod invertebrate guilds) to the stable isotope values in the whole blood of the three *Turdus* thrushes at the Taim Ecological Reserve in southern Brazil, modeled by Bayesian stable isotope mixing models

For the Bayesian stable isotope mixing models, food sources were pooled in categories by similarity according to their ecological characteristics and similar isotopic values. Using credible intervals of 95% (IC_95%_), C3 fruits were the main food source for *T. amaurochalinus* (IC_95%_ = 0.3–0.5, Table 1, Figure 3a) and *T. albicollis* (IC_95%_ = 0.1–0.4, Table 1, Figure 3c), and one of the most important for *T. rufiventris* (IC_95%_ = 0.2–0.3). In addition, arthropod predators were important for *T. amaurochalinus* (IC_95%_ = 0.1–0.4) and were the main food source for *T. rufiventris* (IC_95%_ = 0.5–0.6).

**TABLE 1:** Credible intervals (CI) of 95% of the contributions of potential dietary sources (arthropod invertebrate guilds) for the whole blood synthesis of three *Turdus* species sampled at the Taim Ecological Reserve in southern Brazil

Sources/Birds	Omnivorous	Predators	Herbivorous	Surface‐detritivorous	Terrestrial‐detritivorous	C4/CAM Fruits	C3 Fruits
*T. amaurochalinus*	0–0.12	0.18–0.45	0–0.16	0–0.15	0–0.11	0–0.03	0.36–0.57
*T. albicollis*	0–0.23	0.05–0.38	0–0.33	0–0.3	0–0.22	0–0.06	0.12–0.46
*T. rufiventris*	0–0.05	0.55–0.69	0–0.06	0–0.07	0–0.06	0–0.0	0.21–0.37

**FIGURE 4:**
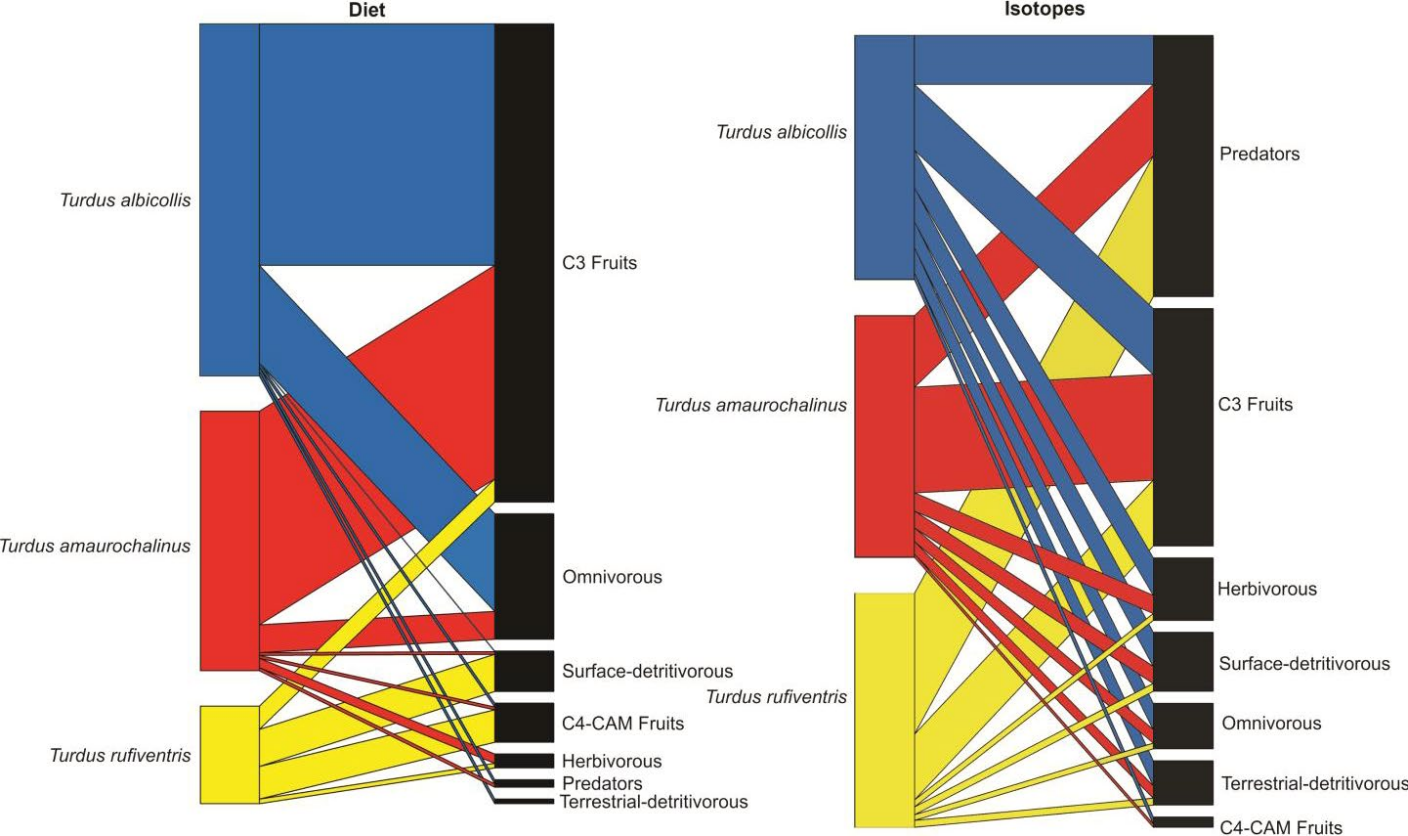
Food‐consumer bipartite network for three *Turdus* species and their food items in southern Brazil. Links represent the consumption of a resource (right column) by each species (left column)

From the resource‐consumer network analysis, nestedness pattern was found between food items and all *Turdus* species (Figure 4), both for the diet (Diet WNODF = 28.1, IC_95%_ = 36.8–68.9) and isotopic networks (Isotope WNODF = 31.9, IC_95%_ = 14.5–31.2). The index *d*′ showed values closer to 0 than 1, demonstrating that the three thrush species are generalists for both interaction networks. In the diet network, *T. amaurochalinus* had *d*′ = 0.09, *T. albicollis d*′ = 0.17, and *T. rufiventris d*′ = 0.56. For the isotopic networks, values were *d*′ = 0.02, 0.1, and = 0.1, respectively.

When analyzing the diet network structure, C3 plants were the food items with the greatest contribution to all three thrush species (Figure 4). For the networks based on the output of isotopic mixing models, invertebrate predators were also highly important, whereas for the dietary network, they were not key food items. The C4/CAM fruits were food items with the lowest contribution to the isotopic networks, while the diet network consisted of terrestrial‐detritivorous invertebrates. The network based on the diet data for *T. rufiventris* had its food items more evenly distributed, failing to demonstrate the importance of invertebrate predators, omnivores, and terrestrial‐detritivores. Notwithstanding, omnivorous arthropods and C3 plants were the most important food items for the diet network of *T. albicollis* and *T. amaurochalinus*. Overall, the networks based on diet and stable isotopes were highly congruent (Figure 4).

## 4 DISCUSSION

As hypothesized, we demonstrated through complementary approaches, high niche overlap between the three thrush species, which are phylogenetically close relatives and morphologically similar species and have similar diets in the forest fragments studied. The greatest trophic and isotopic niche overlap occurred between *T. amaurochalinus* and *T. albicollis*, and was likely due to a shared preference for the fruit of *F. cestrifolia*, which was not present in the diet of *T. rufiventris,* despite low number of diet samples analyzed. While *T. albicollis* lives predominantly in forests, *T. amaurochalinus* also forages in open areas. Thus, *T. amaurochalinus* may be increasing its habitats used for foraging, reducing the effect of overlap and facilitating coexistence among species, as expected by the niche overlap theory (Schoener, 1974).

The species with a narrower isotopic and trophic (fecal) niche overlap with the other species was *T. rufiventris*. This narrow niche overlap could potentially also be a result of underrepresentation for the species due to the low number of fecal (*n* = 4) and isotopic (*n* = 8) samples, despite results encompassed a large range of food items and isotopic values. Furthermore, this low overlap can occur because this species lives in border areas and is often observed feeding in open areas, including places with strong anthropic alterations, such as backyards and gardens; when inside forests, it is seen foraging in the understory or on the ground (Belton, 1985). This preference for forest edges has been demonstrated by its high occurrence and foraging activities in the forest‐open transition area (da Silveira et al., 2016). Thus, *T. amaurochalinus* and *T. rufiventris* forage in areas avoided by *T. albicollis*, a species usually found inside forests only (Belton, 1985). In a study with marked individuals in a mosaic of forest‐open areas, *T. albicollis* was detected inside forested patches only, while *T. rufiventris* and *T. amaurochalinus* were recaptured at forest sites other than those where they were marked, suggesting between‐patch movements (Gasperin & Pizo, 2009).

The trophic overlap between *T. rufiventris* and *T. amaurochalinus* was high (>0.6), while the isotopic overlap was <1%. This difference between the two techniques may have occurred because there is a difference in the time scale between SI and diet methods: While diet reflects feeding up to a few hours before sampling, stable isotope analysis in the whole blood of birds reflects the assimilated diet over a time window of 3–4 weeks (Peterson & Fry, 1987). In addition, fecal analysis has limitations, detecting foods that are hard to digest, thus favoring food items with easily diagnosable hard remains and potentially biasing diet composition information (Ralph et al., 1985). In feces, hard structures predominated, such as exoskeletons of arthropods and seeds, while the stable isotope analysis represents effectively assimilated food items, including those that are easily digested, such as fruit pulp, slugs, and earthworms. Thus, birds may be moving and consuming foods that did not appear in the feces analysis and may not have been considered in the isotopic mixing models. Because thrushes are generalist species, as demonstrated in this study and elsewhere (e.g., Manhães et al., 2010), consumption of a much wider variety of food items than detected in the fecal analysis probably occurred.

Both the fecal and SI approaches indicated a frugivorous pattern for the three thrush species, showing that in addition to being consumed as an energetic resource, proteins in fruits are assimilated by bird tissues. The isotopic results also showed the importance of invertebrate predators as a food source for thrushes, corroborating the results of Lopes et al. (2016) based on diet, and indicating the potential of classification derived from isotopic data. Both approaches would indicate *T. rufiventris* and *T. amaurochalinus* as frugivorous secondarily insectivorous species and *T. albicollis* as a frugivorous species.

The studied thrushes had a frugivorous diet, even presenting a great variety of resources in the fecal samples, but plants were the most important food items, both in the fecal analysis and isotope mixing models. Ruhl, Flaherty, and Dunning (2020) highlights the difficulty of distinguishing isotopic signatures of fruits in bird diets, emphasizing the importance of fruits in the analyses presented here, which highlight the need for well‐supported mixing models. Several studies have indicated that thrushes have fruit‐feeding preferences (Nazaro & Blendinger, 2017; Sabino et al., 2017), but even when predominantly frugivorous, invertebrates are also part of the diet (Gasperin & Pizo, 2009). Thus, the classification of both species as FR_IN_ in this study corroborates the range of studies cited above. In contrast, the most abundant food item for *T. albicollis* was Hymenoptera, even with the classification indicating the species as frugivorous. However, the numerical contribution represents the number of each item recorded in feces, and thus, even with Hymenoptera having a high PN%, these invertebrates are small in size, with a low volume in the samples. When using the PSIRI%, an index that take into account volume and frequency of occurrence, in addition to the numerical frequency (Brown et al., 2012), *T. albicollis* had *F. cestrifolia* fruits emerge as the most important item. As such, stable isotope analysis, which reflects an assimilated diet, seems to demonstrate that some prey/food numerically abundant or frequent but small in size did not contribute substantially to tissue synthesis in birds.

Studies using stable isotopes for the construction of consumer‐food bipartite networks involving birds are scarce (e.g., Dalerum et al., 2016; Miranda et al., 2014). In both cited studies, raw isotopic values were used for the construction of the interaction networks, grouping resources into categories according to the average of the credible intervals of the isotopic values. Links were represented by the number of consumers who contributed to a given category, which resulted in two networks, each with a separate stable isotope element. Because our goal was to compare the diet analysis with the SI analysis, another approach was used (see the Section 2), that is, the output of the Bayesian mixing models. The use of mixing models allowed the depiction of the contribution of each source to the diet of each species based on the simultaneous use of δ^13^C and δ^15^N values, and, more importantly, showing groups of resources formed according to ecological similarities.

As an additional contribution, the current approach based on the construction of networks using the same categories from the dietary method for grouping food items in the stable isotope analysis approach allowed a direct comparison between networks derived from feces and SI analyses. From another point of view, because diet analysis and stable isotopes have different taxonomic resolutions in terms of food items/sources, building comparative networks makes it possible to test food items effectively assimilated and thus important to consumers. Based on the networks, index *d*′, and the nesting pattern, it was demonstrated that thrushes consume almost all food categories included in the networks, again confirming thrushes as generalists birds. C3 fruits in the current study were one of the most important items in the networks, with strong links to all three thrushes. This was the item with the highest contribution in the diet‐based network and one of the most important in the analysis of isotopic‐based networks, emphasizing frugivory in these species, as demonstrated by the diet and isotopic methods, and reported in previous studies (Nazaro & Blendinger, 2017; Sabino et al., 2017). Our results also suggest that thrushes are key species in the trophic networks of *restinga* forests because they are involved in multiple interactions (Scherer, Maraschin‐Silva, & Baptista, 2007), ingest a large variety of fruits (Scherer et al., 2007, this study), and are common species in these ecosystems (Bispo & Scherer‐Neto, 2010; Harrison, Whitehouse, & Madureira, 2013). The C4/CAM fruits contributed a small amount in networks, with the only exception of *T. rufiventris*. This shows that consuming these fruits may not be important for tissue synthesis due to its low contribution to the isotope network, despite they could be important as energetic resources used ready for catabolism. Fruits are intrinsically difficult to distinguish based on isotopic values of fruits in the diet of birds, especially with the conversion of fruit proteins to consumer tissues, which can influence our results (Ruhl et al., 2020).

In conclusion, the present study demonstrated the partitioning of food resources and niche overlap among three sympatric thrushes in southern Brazil. *Turdus* are morphologically similar species, with very similar diets and variable levels of niche overlap. The results of this study illustrated that thrushes are generalist species, but they have some distinct feeding habits; thus, these species can share their trophic and isotopic niches and coexist. The analysis of the feces, together with the stable isotope analysis, provides a comprehensive and robust complementary approach to niche overlapping studies. In addition, the present study provides a new use of network analysis integrated with stable isotope mixing models, allowing comparisons between traditional dietary analysis and the isotopic approach, which reflects food items effectively assimilated in consumer tissues. We demonstrated the importance of fruits in the analysis of feces, stable isotopes, and bipartite networks based on both data sets. Thus, it is recommended that further studies apply this approach, with other species as models, to understand the patterns of consumer‐resource interactions involving a larger number of consumers.

## CONFLICTS OF INTEREST

The authors declare no conflicts of interest.

## AUTHOR CONTRIBUTION

**Camila Bosenbecker:** Conceptualization (equal); Formal analysis (lead); Investigation (equal); Methodology (equal); Visualization (equal); Writing‐original draft (lead); Writing‐review & editing (equal). **Leandro Bugoni:** Conceptualization (equal); Formal analysis (supporting); Funding acquisition (lead); Investigation (equal); Methodology (equal); Supervision (lead); Writing‐review & editing (equal).

## ETHICAL APPROVAL

Instituto Chico Mendes de Conservação da Biodiversidade (ICMBio) allowed the study to be carried out through License SISBIO No. 54642‐1.

## Supporting information

Table S1‐S2

## Data Availability

Data is available from the Dryad Digital Repository at https://doi.org/10.5061/dryad.np5hqbzqd
