# Supplementary material for: Trophic niche similarities of sympatric Turdus thrushes determined by fecal contents, stable isotopes, and bipartite network approaches
Source: Ecol Evol. 2020 Aug 17;10(17):9073–84. doi: 10.1002/ece3.6485 (PMC7487231; doi:10.1002/ece3.6485)
Supplement: Supplementary file 1 — Table S1‐S2 [file ECE3-10-9073-s001.doc]

**Supplementary material**

**TABLE S1** Diet composition of the three *Turdus* species at the Taim Ecological Reserve, southern Brazil, determined through faecal analysis. n = number of faecal samples collected. Frequency of occurrence (FO%), prey-specific numeric contribution (PN%), relative prey-specific volume contribution (PV%), and prey-specific relative importance index (PSIRI%).

| Taxa | *T. amaurochalinus* (n = 26) | | | | *T. albicollis* (n = 20) | | | | *T. rufiventris* (n = 4) | | | |
| --- | --- | --- | --- | --- | --- | --- | --- | --- | --- | --- | --- | --- |
|  | FO% | PN% | PV% | PSIRI% | FO% | PN% | PV% | PSIRI% | FO% | PN% | PV% | PSIRI% |
| **Arthropoda** | 69.23 | 41.05 | 11.55 | 18.21 | 45.00 | 43.69 | 9.03 | 11.86 | 50.00 | 21.05 | 17.78 | 9.71 |
| **Chelicerata** | - | - | - | - | 10.00 | 2.91 | 0.80 | 0.19 | - | - | - | - |
| Araneae | - | - | - | - | 5.00 | 1.94 | 0.37 | 0.07 | - | - | - | - |
| Scorpiones | - | - | - | - | 5.00 | 0.97 | 0.43 | 0.04 | - | - | - | - |
| **Crustacea** | 11.54 | 3.16 | 0.38 | 0.21 | 10.00 | 6.80 | 2.08 | 0.44 | - | - | - | - |
| Decapoda | 11.54 | 3.16 | 0.38 | 0.21 | 10.00 | 1.94 | 0.83 | 0.16 | - | - | - | - |
| Isopoda | - | - | - | - | 5.00 | 4.85 | 1.25 | 0.11 | - | - | - | - |
| **Hexapoda** | 61.54 | 37.89 | 10.78 | 14.98 | 40.00 | 33.98 | 6.14 | 8.02 | 50.00 | 21.05 | 17.78 | 9.71 |
| Insecta NI | 42.31 | 12.63 | 1.70 | 2.81 | 20.00 | 3.88 | 1.78 | 0.76 | 25.00 | 5.26 | 1.75 | 1.00 |
| Larvae | 15.38 | 9.47 | 5.89 | 0.71 | - | - | - | - | - | - | - | - |
| Isoptera | 3.85 | 5.26 | 0.02 | 0.04 | - | - | - | - | - | - | - | - |
| Blattodea | - | - | - | - | 5.00 | 0.97 | 0.19 | 0.04 | 25.00 | 5.26 | 2.56 | 0.58 |
| Coleoptera | 11.54 | 3.16 | 0.94 | 0.15 | - | - | - | - | 25.00 | 5.26 | 12.82 | 1.86 |
| Hymenoptera | 15.38 | 6.32 | 2.21 | 0.61 | 25.00 | 29.13 | 4.17 | 2.11 | - | - | - | - |
| Lepidoptera | 3.85 | 1.05 | 0.01 | 0.02 | - | - | - | - | - | - | - | - |
| Diptera | - | - | - | - | - | - | - | - | 25.00 | 5.26 | 0.64 | 0.34 |
| **Plants** | 88.46 | 49.47 | 71.26 | 53.40 | 85.00 | 46.60 | 60.96 | 45.71 | 100.00 | 68.42 | 44.72 | 56.57 |
| Plant fragments NI | 46.15 | 12.63 | 33.87 | 13.49 | 25.00 | 4.85 | 10.98 | 2.72 | 75.00 | 15.79 | 35.41 | 28.12 |
| Seed NI | 3.85 | 1.05 | 0.05 | 0.02 | 5.00 | 22.33 | 3.46 | 0.21 | 25.00 | 42.11 | 2.56 | 2.40 |
| **Caryophyllales** |  |  |  |  |  |  |  |  |  |  |  |  |
| *Opuntia vulgaris* | 3.85 | 1.05 | 2.00 | 0.06 | 5.00 | 0.97 | 3.33 | 0.09 | 25.00 | 5.26 | 0.13 | 0.80 |
| **Sapindaceae** |  |  |  |  |  |  |  |  |  |  |  |  |
| *Allophylus edulis* | 3.85 | 1.05 | 1.02 | 0.04 | - | - | - | - | - | - | - | - |
| **Myrtales** |  |  |  |  |  |  |  |  |  |  |  |  |
| *Myrsine* sp. | 23.08 | 20.00 | 10.81 | 2.48 | 20.00 | 8.74 | 13.70 | 2.38 | 25.00 | 5.26 | 6.63 | 1.61 |
| **Moraceae** |  |  |  |  |  |  |  |  |  |  |  |  |
| *Ficus cestrifolia* | 50.00 | 13.68 | 23.52 | 11.31 | 50.00 | 9.71 | 29.48 | 13.51 | - | - | - | - |
| NI | 34.62 | 9.47 | 17.58 | 4.36 | 45.00 | 9.71 | 30.02 | 11.39 | 50.00 | 10.53 | 37.50 | 14.06 |

**TABLE S2** Isotopic values of potential food items used in the Bayesian isotope mixing models and mean isotopic values in the blood of the three species of thrushes (*Turdus* spp.) sampled at the Taim Ecological Reserve, southern Brazil, in 2016 and 2017. For isotopic niche ellipses, mixing models and bipartite network analyses, bootstrap values with n = 50 were used for all thrushes

| Taxa (sample size n) | Mean ± SD *δ*^13^C (‰) | Mean ± SD *δ*^15^N (‰) |
| --- | --- | --- |
| **Birds** |  |  |
| *Turdus amaurochalinus* (36) | -23.4 ± 1.5 | 10.0 ± 1.5 |
| *Turdus albicollis* (21) | -24.4 ± 1.1 | 10.4 ± 1.3 |
| *Turdus rufiventris* (8) | -25.0 ± 0.7 | 11.6 ± 1.0 |
| **Potential food sources** |  |  |
| **Omnivorous** | **-23.21 ± 1.98** | **8.36 ± 0.4** |
| Hymenoptera: Formicidae sp. 1 (2) | -21.81 ± 0.4 | 8.02 ± 0.1 |
| Hymenoptera: Formicidae sp. 2 (1) | -24.62 | 8.70 |
| **Predators** | **-26.13 ± 1.3** | **9.82 ± 1.0** |
| Odonata (1) | -27.11 | 9.09 |
| Araneae (2) | -25.15 ± 0.1 | 10.56 ± 0.08 |
| **Herbivorous** | **-25.15 ± 2.6** | **6.67 ± 3.1** |
| Hemiptera: Pentatomidae (1) | -27.55 | 2.27 |
| Lepdoptera (larvae) (2) | -20.08 ± 6.6 | 7.62 ± 4.4 |
| Orthoptera (3) | -26.72 ± 0.3 | 4.68 ± 0.1 |
| Hemiptera: Reduviidae (3) | -24.38 ± 0.1 | 8.93 ± 0.3 |
| Diptera: Tipulidae (3) | -26.36 ± 0.06 | 9.68 ± 0.08 |
| **Surface detritivorous** | **-25.17 ± 0.2** | **6.64 ±1.7** |
| Coleoptera: Scarabaeidae (1) | -25.07 | 4.71 |
| Blattodea (3) | -25.50 ± 1.9 | 7.08 ± 2.3 |
| Isoptera (3) | -24.93 ± 0.2 | 8.11 ± 0.3 |
| **Detritivorous of soil** | **21.82 ± 3.3** | **10.39 ± 1.8** |
| Coleoptera (larve) (2) | -24.22 ± 0.6 | 11.68 ± 0.6 |
| Annelida (4) | -19.42 ± 5.2 | 9.10 ± 1.1 |
| **C4/CAM Fruits** | **-13.35 ± 0.17** | **5.88 ± 2.1** |
| *Bromelia antiacantha* (3) | -13.18 ± 0.1 | 3.78 ± 0.5 |
| *Cereus uruguayanus* (3) | -13.54 ± 0.1 | 5.83 ± 1.3 |
| *Opuntia vulgaris* (3) | -13.33 ± 0.06 | 8.04 ± 0.4 |
| **C3 Fruits** | **-28.66 ± 1.2** | **6.11 ± 1.9** |
| *Passiflora* sp. 1 (3) | -26.75 ± 0.2 | 7.71 ± 0.7 |
| *Passiflora* sp. 2 (2) | -28.01 ± 0.6 | 6.76 ± 0.9 |
| *Solanum sisymbriifolium* (3) | -29.92 ± 2.2 | 8.64 ± 1.1 |
| *Blepharocalyx salicifolius* (3) | -27.38 ± 0.4 | 8.17 ± 0.3 |
| *Sideroxylon obtusifolium* (3) | -27.80 ± 0.8 | 5.48 ± 1.1 |
| *Allophylus edulis* (3) | -28.39 ± 0.6 | 5.70 ± 1.0 |
| *Eugenia uruguensis* (3) | -28.62 ± 0.4 | 6.63 ± 0.3 |
| *Ficus cestrifolia* (3) | -29.10 ± 0.4 | 4.25 ± 0.3 |
| *Chrysophyllum marginatum* (3) | -29.70 ± 0.2 | 5.49 ± 0.2 |
| *Myrsine* sp. (3) | -30.96 ± 0.1 | 2.29 ± 0.1 |
